# Calcium Phosphate Nanoparticles for Therapeutic Applications in Bone Regeneration

**DOI:** 10.3390/nano9111570

**Published:** 2019-11-06

**Authors:** Tanya J. Levingstone, Simona Herbaj, Nicholas J. Dunne

**Affiliations:** 1 School of Mechanical and Manufacturing Engineering, Dublin City University, Dublin 9, Ireland; tanya.levingstone@dcu.ie (T.J.L.); simona.herbaj2@mail.dcu.ie (S.H.); 2 Centre for Medical Engineering Research, School of Mechanical and Manufacturing Engineering, Dublin City University, Stokes Building, Collins Avenue, Dublin 9, Ireland; 3 Tissue Engineering Research Group, Royal College of Surgeons in Ireland, Dublin 2, Ireland; 4 Trinity Centre for Biomedical Engineering, Trinity Biomedical Sciences Institute, Trinity College Dublin, Dublin 9, Ireland; 5 School of Pharmacy, Queen’s University Belfast, Belfast BT7 1NN, UK; 6 Department of Mechanical and Manufacturing Engineering, School of Engineering, Trinity College Dublin, Dublin 2, Ireland; 7 Advanced Materials and Bioengineering Research Centre (AMBER), Royal College of Surgeons in Ireland and Trinity College Dublin, Dublin 2, Ireland

**Keywords:** bone tissue engineering, calcium phosphates, drug delivery, gene therapy, nanoparticle, non-viral vectors, therapeutic delivery

## Abstract

Bone injuries and diseases constitute a burden both socially and economically, as the consequences of a lack of effective treatments affect both the patients’ quality of life and the costs on the health systems. This impended need has led the research community’s efforts to establish efficacious bone tissue engineering solutions. There has been a recent focus on the use of biomaterial-based nanoparticles for the delivery of therapeutic factors. Among the biomaterials being considered to date, calcium phosphates have emerged as one of the most promising materials for bone repair applications due to their osteoconductivity, osteoinductivity and their ability to be resorbed in the body. Calcium phosphate nanoparticles have received particular attention as non-viral vectors for gene therapy, as factors such as plasmid DNAs, microRNAs (miRNA) and silencing RNA (siRNAs) can be easily incorporated on their surface. Calcium phosphate nanoparticles loaded with therapeutic factors have also been delivered to the site of bone injury using scaffolds and hydrogels. This review provides an extensive overview of the current state-of-the-art relating to the design and synthesis of calcium phosphate nanoparticles as carriers for therapeutic factors, the mechanisms of therapeutic factors’ loading and release, and their application in bone tissue engineering.

## 1. Introduction

Bone defects or loss of bone, whether caused by trauma, congenital disorders or diseases, represent a significant burden for the population and the health system. The ability of bones to self-heal depends mainly on three factors: (1) The size of gap to bridge, (2) the stability of the fracture site and (3) the patient’s bone quality. Many common diseases can affect the quality and self-healing potential of bones, above all osteoporosis [1]. Other factors playing a significant role in bone quality include older age and diabetes. When considering that an average of 10 million people are affected by osteoporosis and/or diabetes in the US, and the ageing population, the challenges relating to bone regeneration are expected to continue to rise in the future [2]. Therefore, there is an increasing need for the development of effective therapies for bone regeneration.

In order to address this clinical challenge, there has been an increased interest in the development and administration of therapeutic factors to promote bone tissue regeneration. Generally, these therapies are delivered systemically which has numerous disadvantages including the requirement for larger doses and the potential for off-target effects. Therefore, research has focussed on the development of biomaterials that can act as carriers for the localised targeted delivery of drugs, therapeutic factors and genetic cargoes that can treat diseases and promote bone healing and thus overcome some of the limitations associated with systemic delivery. Among the biomaterials being considered to date, calcium phosphates, such as hydroxyapatite (HA), have been extensively investigated for use in bone repair applications due to their similarity to the mineral phase of natural bones, which confers them an excellent biocompatibility [3]. Many calcium phosphates are also osteoinductive as the high levels of calcium and phosphate ions effectively enhance the osteogenic differentiation of pluripotent cells into osteoblasts [4,5], and most are considered bioresorbable [6]. The family of calcium phosphates is therefore regarded as a safe and efficient class of material for use in bone repair applications.

**Figure 1:**
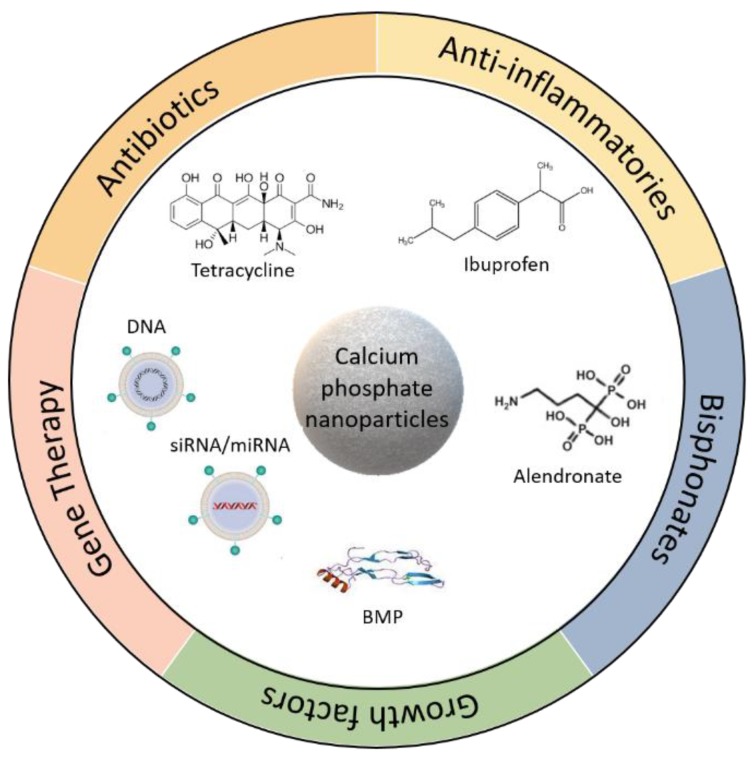
Application of calcium phosphate nanoparticles for the delivery of therapeutic factors for bone repair.

Different approaches have been proposed over the years to tailor the application of calcium phosphate-based materials to the bone healing process. The current trend is focussed on the design and development of calcium phosphates in nanoparticulate form as it has been reported that hydroxyapatite nanoparticles best replicate the form of calcium phosphate found in natural healthy bones [5]. Nanoparticles employed for therapeutic applications are generally between 10 to 100 nm in size, as smaller particles are easily excreted by the kidneys, and particles of a large size are usually removed by the spleen after being phagocytosed [7]. Such nanoparticles have been found to offer a number of advantages in relation to their therapeutic applications in vivo: They are generally well-accepted by the body and have a large surface-to-volume ratio that allows for a higher driving force for diffusion and increased particle solubility [8]. This high surface-to-volume ratio can influence the adhesion of specific proteins, making them particularly suited for the delivery of therapeutic factors [8]. Calcium phosphate nanoparticles have been successfully used for the delivery of a range of therapeutic factors for bone repair (Figure 1), some of which include antibiotics [9], anti-inflammatory agents and growth factors, such as bone morphogenetic factors (BMPs) and cytokines in order to enhance osteogenesis [10]. They have also shown promise for use in conjunction with gene therapy to deliver therapeutic cues for bone repair purposes, whereby the nanoparticles interact with the host tissue, producing a complex that can further enhance bone tissue repair and regeneration [10,11].

Gene therapy is regarded as a more effective way to deliver osteogenic key factors. The approach makes use of small circular DNA molecules, known as plasmid DNA (pDNA), to deliver specific genes encoding particular proteins. These DNA molecules are then loaded on a delivery vector designed to enhance cellular uptake [10]. Numerous studies have proven the feasibility of using calcium phosphate nanoparticles as delivery vectors for gene therapy, for example using pDNA encoding bone morphogenetic protein-2 (BMP-2) [12]. MicroRNAs [13] and silencing RNA (siRNAs) [14] have also been successfully delivered using calcium phosphate nanoparticles [15]. Furthermore, calcium phosphate nanoparticles loaded with therapeutic factors have been combined with scaffolds and hydrogels in order to deliver them to the site of bone injury within the body [16,17].

## 2. Calcium Phosphates

The family of calcium phosphates (CaPO_4_-based) is commonly characterised based on its chemical composition, crystallinity and morphology [18]. Different types of calcium phosphates can consequently be classified as per Table 1. The main differences between such calcium phosphates relate to their Ca/P ratio and solubility, with HA exhibiting the highest Ca/P ratio and least solubility in a physiological environment. Therefore, the resorption kinetics of calcium phosphates strongly depend on the Ca/P ratio, hence permitting the in vivo bioresorbability to be tailored and allowing the most appropriate phase composition of calcium phosphates to be selected for a particular application [18]. HA, alpha-tricalcium phosphate (α-TCP) and beta-tricalcium phosphate (β-TCP) have been the most widely investigated for bone tissue engineering applications.

HA corresponds to the natural mineral phase present in calcified tissues. In the physiological environment, HA is typically observed as nano-sized rods, 30–50 nm in length, 15–30 nm in width and 2–10 nm in thickness [8]. The application of calcium phosphate-based materials in bone tissue regeneration is supported by the important role played by Ca^2+^ and PO_4_^3−^ ions in the regulation of bone resorption and bone deposition. Healthy bones, indeed, undergo continuous remodelling regulated by osteoclasts, osteocytes and osteoblasts communicating mainly through channels of Ca^2+^ and ${\mathrm{PO}}_{4}^{3-}$ ions. While most calcium phosphate-based materials are osteoconductive, some are also osteoinductive, therefore inducing undifferentiated cells to differentiate along the osteoblast lineage [19]. For example, Liu et al. showed that incorporating α-TCP to bone marrow stromal cells (BMSCs) can enhance the expression of key osteogenic proteins, such as runt-related transcription factor 2 (RUNX2), SP7 transcription factor, alkaline phosphatase (ALP) and collagen type I (ColI) [20].

HA is the most stable of the CaPO_4_-based family, exhibiting a stoichiometric formula of Ca_10_(PO_4_)_6_(OH)_2_ and a Ca/P ratio of 1.67. However, HA is not soluble under in vivo conditions [21]. α-TCP and β-TCP have a Ca/P ratio of 1.50, but differ with respect to their crystal structures, which in turn affects their thermodynamic behaviour and dissolution rates in vivo, with α-TCP degrading at a faster rate than β-TCP [22]. In terms of in vivo host response, CaPO_4_ materials are regarded as both bioactive and bioresorbable, depending on their structure, crystallinity and Ca/P ratio. For example, a highly dense and crystalline HA will remain in the body for five to seven years before any physical change is noticeable, whereas, a highly porous HA or a HA nanoparticle powder can be resorbed in less than one year [18]. The in vivo response to calcium phosphate-based particles is further influenced by factors including particle size, shape, porosity and surface area, which play an important role in dictating the cell-material interactions that occur [18].

The use of calcium phosphate nanoparticles as carriers for drugs, therapeutic factors and genetic cargoes has been shown to be an effective approach for the therapeutic treatment of bone diseases and injuries. They have been reported to be less toxic than silica, quantum dots, carbon nanotubes or magnetic particles and more stable than liposomes, thus enabling a more controlled and reliable drug delivery [23]. Characteristics of these nanoparticles, including crystallinity, microstructural properties, surface area, particle size, morphology and surface charge, influence their drug adhesion and ultimately delivery behaviour [24]. In terms of crystallinity, higher levels of drug adsorption have been observed on HA nanoparticles with lower crystallinity [25]. It has been proposed that less crystalline materials exhibit more reactive surfaces and this higher chemical reactivity in the region between neighbouring grains facilitates enhanced drug adsorption [25,26]. The crystallinity of the calcium phosphate particles also affect their solubility and hence the rate of drug release, with lower release rates observed from particles of higher crystallinity [27]. Protein adsorption has also been shown to be dependent on the specific surface area of HA particles [27]. Iafisco et al. reported higher levels of drug complex loading for HA nanoparticles characterised by lower crystallinity and higher surface area, compared to similar nanoparticles of higher crystallinity [28].

Due to the structural flexibility of calcium phosphate-based materials, they can be produced with a range of morphologies including rods, spheres, needles, whiskers and platelets [29]. Palazzo et al. compared the efficiency of drug loading on plate-like and needle-like HA nanoparticles and reported that the adsorption of therapeutic drugs was approximately 1.3 times higher in plate-like HA particles than needle-like particles [30]. Uskoković et al. reported that calcium phosphate nanoparticles with a spherical particle size provided more effective drug loading and drug release properties than particles demonstrating flaky, brick-like or elongated orthogonal morphology [31]. Mesoporous calcium phosphate nanoparticles, which are characterised by a pore size between 2 nm and 50 nm, have recently received great attention because of their improved surface area for drug delivery. Chen et al. successfully produced HA nanoparticles with a uniform mesoporous structure by adding mono-alkyl phosphate to the hydrothermal process and optimising the temperature of the reaction, and demonstrated their improved potential for drug delivery [32]. Particle surface charge is also an important consideration as negatively charged proteins bind more readily to positively charged substrates [30].

In addition to influencing drug loading and release rates, the cellular response to calcium phosphate particles is also influenced by properties such as their particle size and crystallinity. Particles with high crystallinity and low solubility have been shown to promote cell adhesion [33]. A number of studies have shown that calcium phosphate nanoparticles can enhance cell proliferation and mineralisation, with 20 ± 5 nm being the size best accepted by osteoblast cell lines [8]. Furthermore, calcium phosphate nanoparticles are easily endocytosed by cells and have a high binding affinity to various molecules, which provides particular advantages for their application as non-viral vectors for gene delivery for bone repair applications [34]. The morphology of calcium phosphate nanoparticles can be altered to maximise cellular uptake. In addition to the beneficial physical and biological properties, calcium phosphate nanoparticles offer further advantages that support their use in bone repair applications, which include low cost, adaptability to large-scale production, high reproducibility and non-immunogenicity.

## 3. Drug and Therapeutic Factor Delivery

The use of calcium phosphate nanoparticles for the delivery of a range of therapeutic factors has been explored, including low-molecular-weight drugs (e.g., antibiotics and anti-inflammatory drugs) and high-molecular-weight agents (e.g., growth factors) that influence bone healing processes [35]. The development of dual delivery calcium phosphate nanoparticle systems has also been explored, whereby more than one therapeutic factor is delivered in order to achieve a more efficacious healing response. For example, Madhumathi et al. used calcium phosphate nanoparticles for the co-delivery of the antibiotic, tetracycline and the anti-inflammatory, ibuprofen [36]. Bisso et al. used a similar approach for the dual delivery of bisphosphates and nucleic acids [37]. Therapeutic factors can be loaded onto calcium phosphate particles either during the synthesis of the calcium phosphate through co-precipitation [38,39,40] or by combining with the calcium phosphate powders post-synthesis through absorption processes [36]. Co-precipitation can achieve strong chemical interactions between the therapeutic factor and calcium phosphate. Absorption of the therapeutic factor can result in either physical (physisorption) or chemical (chemisorption) interactions between the particle and therapeutic factor. Physisorption results in the binding of the therapeutic factor to the calcium phosphate surface via weak non-covalent bonding, such as hydrogen bonding, hydrophobic interactions, Van der Waals and/or electrostatic forces [25]. Chemisorption involves the formation of new chemical bonds between the therapeutic factor and the calcium phosphate particles. The release of therapeutic factors from calcium phosphate particles has been shown to occur through: (1) Diffusion and leaching of the drug molecule and (2) erosion of the particles through progressive degradation [25]. The release mechanism and release rate are dependent on the level of interaction between the therapeutic factor and the calcium phosphate particles. Physisorption mainly results in the release of the therapeutic factor through diffusion and leaching with release occurring more rapidly than for chemisorption. An initial burst release of the therapeutic factor is often observed when release happens through diffusion and leaching mechanisms [41]. Drugs that are strongly bound to calcium phosphate particles through chemisorption tend not to exhibit a burst release and therefore demonstrate a slower release rate. However, one disadvantage of chemisorption interactions is that the strong bond formed may not be reversible and thus the loaded drug may not be fully recoverable [25].

### 3.1. Antibiotics

Antibiotic treatment is required for bones that are infected, a disease known as osteomyelitis, or at risk of infections, such as after surgery or a penetrating trauma. Bone infections are often caused by bacteria such as Staphylococcus aureus (especially in children), Escherichia coli (E. coli) or Pseudomonas aeruginosa. Such infections are traditionally treated with systemic administration of antibiotics, orally or parenterally [42]. However, antibiotics can have toxic effects on the patients’ metabolism, as high antibiotic quantities are associated with systemic administration. Calcium phosphate nanoparticles have been extensively studied as an antimicrobial approach to reduce infections at the site of injuries, in particular during surgery [43]. The use of ion-substituted calcium phosphates is regarded as a potential approach to sustain and enhance the activity of antibiotic drugs, especially against resistant bacterial strains. Ion-substituted calcium phosphate nanoparticles have been synthesised containing different ions, such as silver [44], zinc [45] and copper [46]. Ion-substitute-based calcium phosphate nanoparticles are normally synthesised using a wet precipitation method [47], microwave synthesis [48] or ion-exchange procedure [49]. However, the use of these metal ions can cause cytotoxic effects. Therefore, a compromise between the antimicrobial effect and the consequent cytotoxic effect has to be considered [43].

To achieve an enhanced antimicrobial effect, various antibiotics including levofloxacin [9], gentamicin, vancomycin, and tetracycline and its derivatives doxycycline, minocycline and tigecycline have been loaded within calcium phosphate nanoparticles. This approach ensures that high concentrations of antibiotics are only found at the anatomical site of interest, hence minimising the toxic effects of antibiotics [50]. Ideally, the release profile of the cargo should match the need for an antibiotic in vivo. Generally for antibiotic release, a quick burst release is required, while limiting the release of the drug over a long period of time in order to avoid the possibility of developing antibiotic resistance [51]. The macro- and micro-structure of nanoparticles can be tuned to allow quick and steady drug release. Antibiotics have been successfully combined with calcium phosphate nanoparticles using both absorption and co-precipitation approaches. The interactions that occur vary depending on the antibiotic used. For example, Uskoković successfully combined vancomycin and ciprofloxacin with both amorphous and crystalline HA nanoparticles and found that vancomycin was released more when amorphous powders were used, whereas ciprofloxacin was released more quickly when using the crystalline powders [40]. Gbureck et al. demonstrated that vancomycin and ofloxacin created only weak bonds with calcium phosphate nanoparticles and reported that the absorption of these antibiotics was dependent on their specific surface area and not composition [49].

Tetracycline, however, is known to have an affinity towards divalent metal ions, such as Ca^2+^ ions [52]. Various sites within the tetracycline structure including the β-diketone system, the enol groups and the amide group can chelate the Ca^2+^ ions [41,52]. Other interactions such as Van der Waals attractions and hydrogen bonding also contribute to the association between tetracycline and calcium phosphate nanoparticles [53]. Mukherjee et al. synthesised tetracycline-loaded calcium phosphate nanoparticles using a co-precipitation method to produce a chelating complex between tetracycline and calcium [54]. The resultant highly membrane-penetrating nanoparticles were able to release tetracycline within tetracycline-resistant E. coli cells. The sustained release of almost 99% of the loaded tetracycline occurred over a seven-day period.

Tadic et al. incorporated gentamicin into nanocrystalline calcium phosphate nanoparticles using both absorption and co-precipitation techniques [38]. Higher gentamicin release rates were observed from gentamicin-loaded particles produced using the absorption technique. No structural or chemical changes to the nanocrystalline calcium phosphate phase were observed as a result of either method of gentamicin incorporation. Uskoković et al. combined clindamycin with calcium phosphate nanoparticles with different morphologies, ranging from planar, brick-shaped, rod shaped to spherical, using an absorption process [31]. They found that spherical particles achieved the optimal drug release profiles and bacterial inhibition, while also achieving the highest bone cell viability and upregulation of osteogenic markers.

In order to deliver antibiotic cargoes, calcium phosphate nanoparticles are frequently embedded in a cement-type matrix. Ghosh et al. successfully incorporated vancomycin and ciprofloxacin with HA nanoparticles using an absorption process and obtained a calcium phosphate cement that demonstrated high biocompatibility with osteoblastic cell lines and a strong effect against Staphylococcus aureus, the main bacteria causing osteomyelitis [55]. Ghosh et al. were able to tune the drug release kinetics within a specific period up to 15 days, by tailoring HA nanoparticle size and weight ratio [55].

### 3.2. Anti-Inflammatory

Anti-inflammatory drugs, also known as non-steroidal anti-inflammatory (NSAIDs), are frequently used for the treatment of chronic bone diseases and when pain or inflammation are exhibited. These conditions include osteoarthritis, rheumatoid arthritis and post-operative pain. NSAIDs are used to reduce inflammation, to prevent fever by acting as anti-pyretic drugs, and to provide an analgesic effect [51]. NSAIDs alleviate inflammation by regulating some of the various cytokines and growth factors involved in the activation of the inflammatory tissue response. During inflammation, the expression and proliferation of osteoclasts are particularly enhanced, hence relating bone resorption to the inflammatory response [56]. Osteoclast differentiation is regulated by receptor activator of nuclear factor kappa-B ligand (RANKL), a protein directly correlated to bone formation; tumour necrosis factor alpha (TNF), a cytokine involved in chronic and acute inflammation; and the cytokines family, interleukin-1 (IL-1) [56]. The control over the expression of the inflammatory molecules can be used to encourage the recruitment of mesenchymal stem cells (MSCs) and the initiation of the healing cascade.

The treatment of cultured osteoblasts with different steroids and NSAIDs has shown that the use of corticosteroids is related to the suppression of bone genes, such as runt-related transcription factor 2 (RUNX2), BMP-2 and osteocalcin (OCN), limiting the osteogenesis ability of MSCs [57]. Chang et al. also report that treatment with dexamethasone, an NSAID drug, is related to the inhibition of proliferation of MSCs, while not affecting the expression of such osteogenic genes on an 8-day treatment basis [57]. In other cases, NSAIDs have also been correlated with decreased cell proliferation and delayed fracture healing [58].

The successful delivery of anti-inflammatories using calcium phosphate particles has been demonstrated by numerous authors [36,59]. The delivery of ibuprofen using β-TCP particles was demonstrated by Baradari et al. [59]. Ibuprofen was soak-loaded onto the particles, with the ibuprofen molecules diffusing into the pores by capillarity and remaining trapped there after solvent removal. The amount of ibuprofen within the calcium phosphate-based particles was found to increase linearly with increasing drug concentration in the absorption solution and an adsorption equilibrium time of one hour was reported. Ibuprofen was rapidly released from the particles, confirming the weak binding affinity between ibuprofen and β-TCP. No structural or chemical modifications were observed from physiochemical characterisation of the ibuprofen-loaded particles indicating that the binding interactions occur through physisorption processes [59].

A dual-delivery calcium phosphate carrier for antibiotic and anti-inflammatory delivery for bone regeneration was developed by Madhumathi et al. [36]. Calcium-deficient HA nanoparticles were used for the delivery of the antibiotic, tetracycline and β-TCP for the delivery of the anti-inflammatory drug, ibuprofen. The combined release of antibiotic and anti-inflammatory drugs appears to influence their release in comparison to individual release profiles. Ibuprofen was released from β-TCP nanoparticles with a burst release over the initial 12-h period that can be attributed to the release of the drug physically adsorbed on the surface of β-TCP nanocarriers followed by a slow release up to 5 days. A more controlled release of tetracycline was observed with the maximum release between 24 and 48 h. The tetracycline and ibuprofen release profiles fit with the Higuchi’s model of diffusion indicating that drug release occurred mainly through diffusion processes. Furthermore, this study demonstrated that this combined drug release profile resulted in significant antibacterial and anti-inflammatory activity in vitro and increased levels of bone formation at 12 weeks compared to controls in vivo in a rat cranial model [36].

### 3.3. Bisphosphonates

Therapeutic drug delivery using calcium phosphate nanoparticles is not only interesting for bone regeneration, but it can also lead to a new intervention for the treatment of bone diseases, such as osteoporosis. Bisphosphonates are a class of drugs that are used to prevent bone loss and are commonly used in the treatment of osteoporosis and other diseases that exhibit bone fragility. Examples include alendronate, pamidronate and zoledronate. The mechanism of action involves inhibition of the bone turnover process. However, despite being considered a relatively safe drug, long-term clinical trials have shown that the use of bisphosphonates, particularly high doses used over a long period of time, could be related to atypical bone resorption [60]. Bone necrosis of the jaw and stress fractures to the femoral shaft post-treatment seem to correlate with the administration of bisphosphonates; however, the interactions between drug and disease are still unclear and often difficult to establish due to co-morbidities of the patients [61,62].

The delivery of bisphosphonates using calcium phosphate nanoparticles has been shown to increase the therapeutic effect of the drug and reduce the side effects to non-targeted areas [63,64]. Bisphosphonate-loaded HA particles have been successfully fabricated using co-precipitation and chemisorption methods [64,65]. The co-precipitation method has been shown to achieve increased efficiency of drug loading compared to chemisorption methods. Stronger interaction forces between the drug and calcium phosphate occur as a result of co-precipitation and these lead to a slower drug release rate than for chemisorption methods [65]. Due to the high binding affinity of bisphosphonates for calcium, the addition of bisphosphonates during the co-precipitation process can result in the formation of a calcium bisphosphonate compound and thus hinders the precipitation of the calcium phosphates. Boanini et al. report that this can be overcome by adding the bisphosphate after HA crystal nucleation and growth has started [65,66]. Other calcium phosphate phases, including β-TCP [36] and octacalcium phosphate (OCP) [67], have also been successfully functionalised with bisphosphonates. The chemisorption method presents an easier route for the fabrication of bisphosphonate-loaded calcium phosphate particles. During the chemisorption process, bisphosphonate binds to the calcium phosphates as a result of polar covalent interactions with calcium atoms on the surface of calcium phosphate particles and the displacement of PO_4_^3−^ species [68]. The association modes that occur have been shown to depend on the calcium phosphate phase used. Combining zoledronate with β-TCP has been shown to result in the formation of calcium zoledronate crystals on the surface of the β-TCP particles. For calcium deficient apatite, Josse et al. reported binding to occur mainly through the displacement of phosphate ions with a quantitative plateau in zoledronate uptake at about 10 wt.% which corresponds to the surface saturation point [68].

Bisphosphonate-loaded calcium phosphate particles have also been used in dual drug delivery approaches. For example, Bisso et al. investigated the dual delivery of plasmid DNA and alendronate using calcium phosphate nanoparticles, with the goal of facilitating cellular internalization of both the compounds and potentially achieving a combined pharmacological effect [37]. The study used a pH-sensitive poly(ethylene glycol)-alendronate conjugate to formulate stable plasmid DNA-loaded calcium phosphate nanoparticles. These particles displayed good transfection efficiency in in vitro studies.

### 3.4. Growth Factors

Growth factors are essential molecules that direct and guide tissue formation. The main growth factors relating to bone formation include the family of BMPs, transforming growth factor-beta (TGF-β), platelet-derived growth factor (PDGF) and vascular endothelial growth factor (VEGF) [69]. Each of these growth factors play a particular role in bone repair processes. Specifically, BMPs stimulate osteogenesis, TGF-β regulates cell growth, differentiation and immune function, PDGF promotes cellular proliferation and VEGF induces angiogenesis. While calcium phosphate nanoparticles have demonstrated the potential to promote osteogenesis even when not loaded with any therapeutic factor, the addition of these factors can further enhance calcium phosphate functionality. Growth factors present a higher complexity as a result of their protein nature, high molecular weight and spatial arrangement. Therefore, when loaded onto calcium phosphate nanoparticles, attention is required to avoid denaturing the protein and reducing its functionality.

The use of calcium phosphate nanoparticles as carriers for the sustained release of BMPs has been demonstrated by numerous researchers [70,71]. BMP-2 can reportedly interact with HA through three functional groups -OH, -NH2 and COO [72]. The water-bridged H-bond plays an important role in this interaction. Based on the different orientations of protein, each might interact with an individual HA crystallite or cooperatively with two or three crystallites. When more than one set of adsorption groups is involved for a certain orientation of protein, a step-wise adsorption–desorption process would result, whereas when only one set is involved, there would be only the key adsorption period [72]. Xie et al. explored the potential of HA nanoparticles for the delivery of BMP-2 and used a radiolabelling process to precisely measure the amount of BMP-2 absorbed [70]. They reported the important role of electrostatic attraction in the binding interactions between the HA nanoparticles and BMP-2. Xie et al. demonstrated that BMP-2 could be continuously released from the HA nanoparticles over a 15-day period in vitro. Zhou et al. demonstrated the role of specific surface area in BMP-2/HA binding, reporting that that HA nanospheres (80–150 nm) loaded with BMP-2 achieved improved loading capability than HA microspheres (75–100 μm). The HA nanospheres also showed an improved release profile, without the initial burst release profile observed for HA microspheres [73]. The improved loading and release properties of these HA nanospheres were demonstrated to enhance the availability of BMP-2, inducing osteogenesis and promoting the repair of bone defects.

While BMPs are critical for enhancing osteogenesis, great attention has been given to the effect of VEGF on bone fracture repair. VEGF stimulates angiogenesis, which is pivotal for the healthy growth of bone tissues. Indeed, the lack of a proper blood supply would lead to a lack of oxygen and nutrients and therefore, tissue necrosis, especially in the case of large bone defects. Furthermore, the presence of a well-connected blood supply can guide osteoblasts differentiation and mineralisation [74]. As a result, there is an increasing interest in the incorporation of VEGF, either alone or in conjunction with BMPs, for the treatment of bone fractures. Wernike et al. co-precipitated VEGF onto biphasic calcium phosphate nanoparticles in order to achieve a sustained release of VEGF in vivo for enhanced vascularisation and bone formation [75].

## 4. Calcium Phosphates for Gene Delivery

In addition to their use for the delivery of drugs and growth factors, calcium phosphates have been widely used as non-viral vectors for gene delivery applications for the delivery of DNA-based and RNA-based genetic cargoes [76]. Gene therapy brings about the possibility for the sustained intracellular production of proteins at the required concentrations, thus offering advantages over protein-delivery based systems. Calcium phosphates hold particular advantages as gene delivery vectors as they remain stable within the hostile extracellular environment in order to protect the molecular cargoes. Calcium phosphate nanoparticles can also be easily endocytosed by cells through the lipid bilayer cellular membrane [77]. Once inside the cell, the release of a genetic cargo from the endocytosed calcium phosphate nanoparticles relies on the ability of the calcium phosphate to dissolve efficiently in the acidic environment that results from lysosome activity [78]. Since endosomal escape is directed by the dissolution behaviour of the carrier, faster dissolution leads to a more rapid increase in osmotic pressure and thus earlier endosome escape [79]. Transfection of MSCs using calcium phosphate nanoparticles has been shown to achieve enhanced osteogenesis following transfection with calcium phosphate nanoparticles compared to polyethylenimine (PEI) which failed to induce robust osteogenesis of MSCs [80]. The advantages of calcium phosphates as non-viral vectors thus far outweigh their limitations, such as low transfection efficacy (about 10–20%) if compared to viral vectors (about 40–50%) [12,81].

### 4.1. DNA

Calcium phosphate nanoparticles can be loaded with a DNA cargo, creating a complex DNA-calcium phosphate (DNA-CaP). By binding the DNA cargo to calcium phosphate nanoparticles, the transfection efficacy of the complex is increased as the nanoparticles protect the DNA molecule while providing a means for crossing the cell membrane. Naked DNA, indeed, would quickly be attacked by lysosomes and nucleases, before reaching the nucleus of the cell [82]. Calcium phosphate nanoparticles can penetrate the cell membrane by endocytosis and release the cargo once in the cytoplasm of the targeted cell, thereby delivering the DNA to the nucleus [78]. Overall, these properties make calcium phosphate nanoparticles ideal vehicles for gene therapy. Calcium phosphate nanoparticles are preferable to cationic polymers, lipids or peptides that often cause an adverse response or that might not be degradable [83].

**Figure 2:**
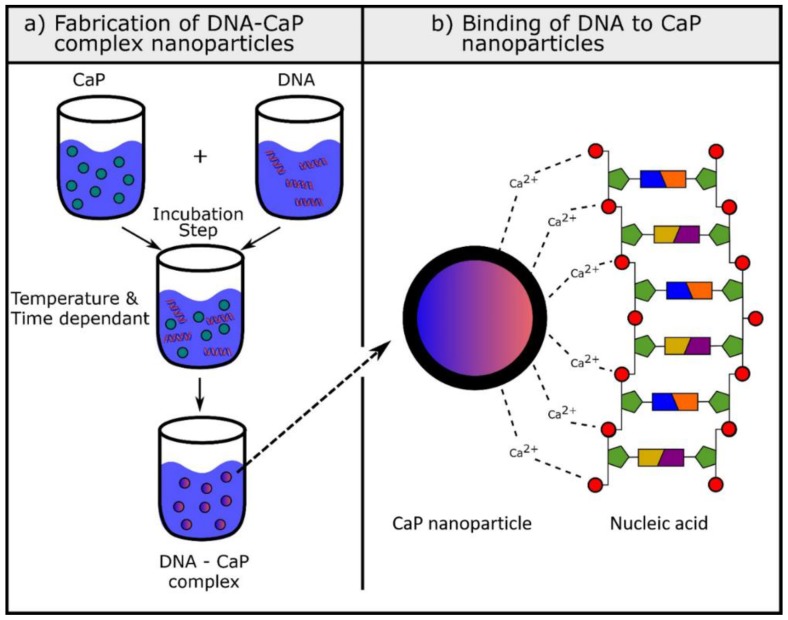
Fabrication of DNA-calcium phosphate (DNA-CaP) complex nanoparticles. (**a**) Shows the fabrication of DNA-CaP complex nanoparticles by co-precipitation method; (**b**) represents a close-up view of the affinity of the phosphate group of the nucleic acid for calcium phosphate nanoparticles.

DNA-CaPs can be manufactured by following different routes, most commonly by using a co-precipitation method where DNA-CaPs spontaneously form in supersaturated solutions (Figure 2a). These nanoparticles are strongly affected by physicochemical conditions and the reaction process therefore must be strictly controlled [11]. When the co-precipitation reaction occurs, the DNA-CaP complex is characterised by the strong adherence of the DNA to the calcium phosphate particles. This is due to the high affinity of calcium phosphates for the phosphate groups of the nucleic acid (Figure 2b). Another approach is to encode the growth factor genes onto plasmid DNA. This method aims to ensure sustained delivery and efficacy of the protein. Epple et al. investigated the transfection efficacy of calcium phosphate nanoparticles loaded with two plasmid DNAs encoding BMP-7 and VEGF-A and reported a transfection efficacy comparable to the commonly used synthetic transfection agent Lipofectamine^®^2000 and cell viability over 90% [84].

One limitation of these systems is that the early degradation of calcium phosphate nanoparticles can often affect transfection efficacy. For this reason, multishell nanoparticles have been developed, which provide a much slower degradation profile and further preserve the DNA cargo [85]. Sokolova et al. successfully developed an approach, which involved preparing a double layer coating of calcium phosphate nanoparticles and a DNA cargo, showing an increased transfection compared to the use of a single layer coating [86]. Multishell nanoparticles also allow for a more sustained controlled release and longer storage time of 2–3 months [43]. Tenkumo et al. fabricated multishell nanoparticles, by combining hydroxyapatite with functionalised pDNA and protamine [87]. These nanoparticles were subsequently loaded in a collagen scaffold for periodontal tissue repair, obtaining high transfection efficacy comparable to Lipofectamine^®^2000 [87].

### 4.2. RNA

RNA interference (RNAi) is a powerful tool for controlling the expression of genes in eukaryotic cells. RNAi comprises of two approaches: (1) microRNA (miRNA) and (2) silencing RNA (siRNA), which bind to specific sites on the messenger RNA (mRNA), hence interfering with the protein expression. miRNAs and siRNAs are naturally occurring non-coding RNAs that play an important role in regulating gene expression. The major difference between them is that siRNAs are highly specific with only one mRNA target, whereas the miRNAs have multiple targets. Therefore siRNAs bind to a single specific gene with a 100% match, whereas miRNAs are able to target hundreds of different genes [88].

#### 4.2.1. MicroRNA

The majority of miRNAs are transcribed from DNA sequences into primary miRNAs and processed into precursor miRNAs, and finally mature miRNAs [89]. miRNAs are directly involved in the regulation of the expression of different genes at post-transcriptional level [90] by binding to reception sites within the cells and guiding cell proliferation, differentiation and death. The interaction of miRNAs with their target genes is dependent on many factors, including the subcellular location of miRNAs, the abundancy of miRNAs and target mRNAs, and the affinity of miRNA–mRNA interactions. miRNAs can stimulate the expression of proteins such as BMPs that are intrinsically related to osteogenesis differentiation. miRNAs are also directly involved in pathways leading to different bone conditions such as osteoporosis and osteoarthritis, as they can upregulate or downregulate genes related to those diseases. Therefore, the possibility to combine miRNAs to delivery vectors, such as calcium phosphates nanoparticles, has attracted significant research attention within the field [91,92].

The miRNAs involved in the regulation of the complex bone metabolism are numerous. They can be broadly divided into two classes, miRNAs influencing osteoblasts proliferation and differentiation by either suppressing the osteogenic differentiation process or by promoting it. One of the most common regulating factors targeted by the miRNA therapeutic approach is the runt-related transcription factor 2, also known as RUNX2, which is heavily involved in the regulation of the expression levels of multiple genes related to bone forming cells. The inhibition of RUNX2 has serious consequences on the ability of cells to differentiate into osteoblasts, which is supported by the notion that mice deficient in RUNX2 lack the formation of skeletal structures, leading to mice death just after birth [93]. RUNX2 can also promote and guide the expression of bone regulating factors, such as OCN, ColI, osteopontin (OPN) and ALP. The expression of genes, such as RUNX2, is regulated by a wide number of miRNAs, which can either promote or suppress the gene. miR-133 is part of those miRNAs that are designed to suppress the expression of RUNX2, hence inhibiting osteogenesis [94]. Therefore, to enhance osteogenesis, it is also possible to tune antagonist miRNAs to downregulate the expression of miRNAs, such as miR-133, in order to ultimately promote the expression of RUNX2. This approach was successfully achieved by Castaño et al., who had incorporated the antagomiR-133a into HA nanoparticles and subsequently loaded the complexes on a collagen scaffold. [95]. The antagomiR-133a could inhibit the activity of miR-133 in human mesenchymal cell cultures, while keeping minimal cytotoxicity [96].

The use of RNAi for the regulation of negative inhibitors is also extensively studied, as in the case of histone deacetylases (HDACs), strong negative inhibitors of RUNX2 [97]. For example, HDAC5 has been shown to be related to RUNX2 degradation, hence decreasing bone formation and accelerating osteoporosis in mice [98]. HDAC5 is transcribed by miR-2861 and in humans, a mutation in pre-miR2861 was found to cause osteoporosis in two adolescents [98]. Other important genes that regulate the expression of proteins involved with bone-forming cells are the transforming growth factor beta (TGF-β) family. TGF-β is particularly important in stimulating the differentiation of MSCs into osteoblasts, through the TGF-β/activin/nodal pathway, which is in turn regulated by the Smad family receptors [99]. Smad-1, Smad-5 and Smad-8 are responsible for the activation of the canonical pathway [97], while Smad-4 serves as a ligand for the Smad receptors, guiding the so-formed complexes towards the nucleus [99]. Some studies on the effect of different miRNAs on bone regulating genes are shown in Table 2. For example, it has been demonstrated that overexpression of miR-206, miR-133, miR-135 and miR125b inhibit osteogenic differentiation. On the other hand, the expression of cluster miR-17-92, miR-196a and miR-2861 has the opposite effect, enhancing osteogenic differentiation [100].

#### 4.2.2. Silencing RNA

Silencing RNAs (siRNAs) are double-stranded RNAs that are widely studied for specific gene silencing. The siRNAs are able to perfectly match specific sites on an mRNA, therefore silencing the expression of specific genes by endonucleolytic cleavage of the mRNA [88] and “knocking-down” the targeted gene. Although the specificity of siRNAs is one of the distinctive features that differentiates it from miRNAs, siRNAs may lead to the downregulation of unintended, unpredicted targets, resulting in off-target effects. One of the major challenges of siRNA therapy is to reduce off-target effects, as these compromise the therapeutic effect and specificity and can even lead to cell death [88]. A number of strategies to overcome this problem have been investigated, including benefitting from siRNA redundancy, applying chemical modification of the siRNAs or eliminating pro-inflammatory sequences to avoid immune-stimulation caused by the siRNAs [104].

A further limitation related to the use of siRNAs is the need to increase cellular uptake by binding siRNAs to a suitable delivery vector. Like in the case of miRNAs, among the many possible delivery vectors, calcium phosphate nanoparticles can be tuned to suit the delivery of siRNAs. Therefore, to enhance loading efficiency, calcium phosphate nanoparticles can be functionalised with amino acids, such as arginine [105]. Nanoparticles composed of encapsulated siRNAs with calcium phosphates can also be stabilised with different coatings, such as PEI [106], natural polymers [107] or biodegradable lipids [105], in order to protect the siRNAs during delivery. An interesting approach investigated for the delivery of siRNAs is the fabrication of multishell calcium phosphate nanoparticles. The idea is to protect the siRNA cargoes against nucleases, while stabilising the complexed nanoparticles. Neuhaus et al. investigated the potential for a triple shell composed of calcium phosphate nanoparticles, siRNAs, and PEI (CaP/siRNA/CaP/PEI) for the delivery of siRNAs to knock down TNF-α for the treatment of tumour cells, and successfully reduced gene expression to 18% of the original value, while cell viability remained over 70% [106]. Similarly, others like Zhang et al. have looked at triple shell calcium phosphates nanoparticles in conjunction with siRNAs and poly-(L-lysine) embedded in a multilayered electrolyte film for control of the expression of OCN and OPN genes over sustained periods [108].

## 5. Delivery Methods

The use of matrices for the delivery of therapeutic factor-loaded calcium phosphate nanoparticles to the site of bone injury or disease has been widely investigated [16]. These matrices provide a template for cell infiltration and tissue formation and therefore can promote an effective bone healing process. They should allow for ease of delivery, support the biological process in vivo, enable the release of calcium phosphate nanoparticles in a controlled manner and permit the transport of drugs and/or miRNAs to the targeted cells [109]. To comply with clinical requirements, the matrix degradation profile should match the natural healing process of bones. The matrix can take the form of solid and porous scaffold or of a hydrogel and can either release its cargo steadily overtime or be tuned to respond to particular stimuli [110,111].

### 5.1. Scaffolds

Some of the basic requirements for bone tissue engineering scaffolds are (1) biocompatibility and bioresorbability, (2) adequate mechanical properties to provide support and (3) an interconnected porous structure [112]. The latter can be a complex requirement to achieve, as an increment in porosity is usually related to a reduction in mechanical strength. Examples of techniques usually employed to obtain such structures include, among many, hot pressing sintering [113], solid freeform fabrication [114] and 3D printing [115]. Recently, selective laser sintering has been used widely as a rapid prototyping technique [116]. By implementing this technique, it is possible to homogeneously add nanoparticles within the scaffolds, such as HA nanoparticles, in a porous β-TCP scaffold [117]. An interesting approach is the one applied by Feng et al., who used selective laser sintering followed by isothermal sintering to obtain a structure with nano grain size and increased density [118]. Alternatively, freeze drying has been used to fabricate highly porous (>98.9%) collagen-based scaffolds loaded with hydroxyapatite nanoparticles [119]. However, the mechanical stiffness of these HA nanoparticle-loaded collagen-based scaffolds is relatively low, which limits their use in non-load bearing applications.

Biomaterials used for producing scaffolds can be divided into natural polymers and synthetic polymers [116]. Collagen is the most widely used natural polymer as it represents the main protein component of the natural structure of most tissues, including bones. However, the use of collagen presents some drawbacks such as the possibility of pathogen transmission and its high cost. Gelatin, a denatured form of collagen is another common natural polymer, which maintains most of the collagen-binding sites, but it is free of immunogenicity [15]. Other natural polymers include chitosan, silk and fibroin. Among the synthetic polymers, poly(lactic-co-glycolic) acid (PLGA), polycaprolactone (PCL) and polylactic acid (PLA) are some of the most common polymers used. They are more versatile than natural polymers, as they can be tailored to achieve a much wider range of properties. Conversely, synthetic polymers either lack biodegradability or when degrading can release toxic by-products.

The advantage of using natural polymers relates to their numerous binding sites, especially sequences of arginine-glycine-aspartic acid (RGD), that cells can easily recognise and interact with [7]. However, natural biomaterials often lack the mechanical strength required for load-bearing bone tissue engineering applications. Adding calcium phosphate nanoparticles is one approach to overcome this limitation. Cunniffe et al. added various amounts of HA nanoparticles homogeneously to a porous collagen scaffold, showing a significant increase in mechanical properties [119]. Another approach investigated electrospinning to fabricate scaffolds with increased mechanical strength, resulting in highly well-oriented fibres. For example, gelatin solutions can be electrospun to increase their Young’s modulus [120]. The gelatin electrospun fibres can also be further crosslinked with UV light [121].

Even though significant improvements have been achieved with increasing the mechanical strength of natural polymers, often this still does not match the natural strength of bones, especially with regards to applications for load-bearing sites. Therefore, natural biomaterials are commonly combined with synthetic polymers. For example, biphasic calcium phosphate scaffolds can be coated with poly-L-lactic acid (PLLA) and HA nanoparticles to obtain a structure with enhanced mechanical properties, whilst still maintaining good porosity and biocompatibility [122]. Similarly, amorphous calcium phosphate and HA nanoparticles can be electrospun on PLA to obtain stronger structures that still support osteogenesis and offer good potential for bone repair in vivo [123]. Calcium phosphate nanoparticles can also be used to guide and enhance osteogenesis on synthetic-based scaffolds, such as the work conducted by Song et al., who incorporated biphasic calcium phosphate on 3D printed PCL constructs, inducing enhanced bone formation in vivo [124].

A number of studies have functionalised scaffolds for bone tissue repair applications through the incorporation of therapeutic-loaded calcium phosphate nanoparticles [12,16,17,38,96]. For example, Tadic et al. fabricated gentamicin-loaded calcium phosphate powders and then used a cold-pressing process to produce cylindrical scaffolds for use as drug-loaded calcium phosphates for the treatment of bone infections [38]. Curtin et al. combined BMP-2-loaded HA nanoparticles with collagen-based scaffolds to create gene-activated matrices for BMP-2 delivery and demonstrated successful transfection of MSCs, resulting in high levels of calcium production [12]. These gene-activated matrix systems were further employed for the dual delivery of VEGF and BMP-2 resulting in increased vascularisation and bone repair in vivo [125]. Functionalisation of collagen HA scaffolds with miR-113a inhibiting complexes delivered using HA nanoparticles has also be reported [96].

### 5.2. Hydrogels

Hydrogels are 3D networks that present high hydrophilicity and biocompatibility, along with the possibility to be delivered in a minimally invasive manner in vivo [126]. Analogously to scaffolds, hydrogels can be composed of natural polymers (e.g., collagen, alginate and fibrin), synthetic polymers (e.g., polyacrylamide (PA) and polyethylene glycol (PEG)) or a combination of both [127]. Hydrogels are often combined with calcium phosphates, as they provide an adhesive, cohesive and malleable network for the delivery of osteoconductive and osteoinductive calcium phosphate nanoparticles, which are often functionalised for the delivery of therapeutic factors as discussed above. Apart from being used for therapeutic delivery, the addition of calcium phosphate nanoparticles can mechanically strengthen the hydrogel structure, as demonstrated both by Pavlov et al., who used TCP to reinforce alginate hydrogels [128] and by Turco et al., who combined alginate hydrogels with HA nanoparticles and obtained a compressive modulus of 83 kPa for alginate-HA hydrogels compared to 20 kPa of HA-free alginate hydrogels [129]. The incorporation of calcium phosphate nanoparticles in hydrogel structures leads to an increase in surface roughness that has been reported to encourage cell attachment [130].

Furthermore, hydrogels can be found in a swollen gel form or they can undergo gelation upon delivery in situ, which makes their use very appealing for the clinical practice [131]. For example, combining alginate with poly(N-isopropylacrylamide) (PNIPAAM) results in a thermo-responsive hydrogel that undergoes gelation once implanted in vivo, at a temperature of approximately 37 °C [132]. Wu et al. developed a thermo-responsive hydrogel containing PNIPAAM loaded with mesoporous calcium phosphate particles and therapeutic drugs in order to offer a sustained release profile for a period of 16 days [133]. Finally, the effect of adding a range of BMP-2-loaded calcium phosphate nanoparticles embedded in a hyaluronic acid hydrogel was studied in vivo by Hulsart-Billström et al. by investigating the extent of ectopic bone formation in rats over a 4-week period [71]. Their study concluded that 20 nm was the optimum particle size of HA nanoparticles for successful delivery of BMP-2, as this group yielded a repair tissue with the highest bone density when compared with the use of micro- or macro-sized HA particles. Their findings are likely due to the ability of the HA nanoparticles to achieve enhanced loading and release of the BMP, while also acting as a “building block” for mineralisation [71].

### 5.3. Stimuli-Controlled Matrices

Ideally, the matrix surrounding complexed-calcium phosphate nanoparticles should provide a template for bone formation, while slowly releasing its cargo. However, the release of therapeutic factors from the matrix is often too fast or difficult to regulate. Therefore, new systems have been explored in order to achieve a more precisely controlled release of a drug when it is needed, “on demand”. Approaches used include the use of magnetic nanoparticles and pH-responsive nanoparticles [134,135,136,137]. Magnetic nanoparticles can been produced by coating their surface with iron oxide and functionalising the particles using ligands to target specific cells [135]. This approach allows magnetic nanoparticles to be tracked in vivo. The particles can also deliver magnetic fields that can be translated into forces sensed by the underlying cells [135]. The excitation of mechano-transduction pathways has been shown to trigger the response of MSCs and to stimulate the production of new bones [138]. Studies have demonstrated that mineralisation can be promoted when magnetic nanoparticles act in conjunction with growth factors (e.g., BMP-2) [134]. Calcium phosphate magnetic nanoparticles have been successfully developed by Puddu et al., where tricalcium phosphate nanoparticles were doped with iron oxide by flame spray synthesis, and demonstrated a good transfection efficacy of about 40%, while maintaining 99% cell viability and a proliferation rate of over 50% [136].

Another approach for the controlled release of therapeutic drugs is the development of pH-responsive calcium phosphate nanoparticles. This has been explored by Zhang et al. using bovine serum albumin as a drug model. Their findings suggest that the release of the protein by mesoporous HA nanoparticles depends on the environmental pH at which the HA nanoparticles absorbed the protein [139]. Calcium phosphates have also been used as coatings for pH-controlled silica nanoparticles for drug delivery, due to the ability of calcium phosphates to dissolve in acidic environments [137].

## 6. Conclusions and Future Perspective

Bone diseases and injuries are becoming more prominent in society, especially since several conditions, such as diabetes, strongly influence the ability of bones to self-repair. The need for efficient therapies constantly guides research towards the investigation of new techniques which could lead to more effective treatments. Current strategies to enhance bone regeneration concentrate on investigating the incorporation of drugs, growth factors or genes to promote and facilitate the natural healing process of bones. Since nanoparticles are highly suited for the incorporation of such cues, it seems likely that the use of nanoparticle-based delivery systems will become more and more prevalent in the coming years. Furthermore, the latest discoveries and advances in gene delivery is leading the way to a new approach for tissue regeneration. In this context, the use of calcium phosphate nanoparticles offers great potential for bone regeneration purposes. Calcium phosphates provide excellent biocompatibility and hence, without incurring adverse body reactions, they also supply a natural source of calcium ions, encouraging bone mineralisation. One of the most interesting feature of calcium phosphate nanoparticles is the possibility to load a variety of drugs on their surface, hence providing a template for the treatment of multiple diseases.

Finally, the potential for calcium phosphate nanoparticles to act as non-viral vectors for gene delivery proposes makes them an ideal platform for the implementation of new strategies for the treatment of bone diseases. Indeed, the increasing interest in non-viral vector platforms for gene therapy is a further reason for the consideration of calcium phosphate nanoparticles as ideal candidates for bone tissue engineering applications. Calcium phosphate nanoparticles have demonstrated the ability to facilitate the delivery of miRNAs and siRNAs, forming stable complexes that can easily be endocytosed by the targeted cells, hence allowing the release of miRNAs and siRNAs directly within the cytoplasm of cells. This characteristic taken in conjunction with the natural biocompatibility and promotion of bone mineralisation on behalf of calcium phosphates suggest that calcium phosphate nanoparticles will be regarded as potential non-viral vector carriers for the treatment of a wide variety of bone diseases. Furthermore, the use of scaffold and hydrogel systems for the delivery of therapeutic factor-loaded calcium phosphate nanoparticles presents an exciting prospect for achieving repair of complex bone injuries and non-union fractures. While such approaches have been explored, the ideal composition for a bone grafting material that can provide the porous structure required for cellular infiltration and sufficient mechanical properties for use in load-bearing applications has not yet been established on a clinical basis. Further efforts should lean towards the understanding of the underlying mechanisms and processes that guide bone formation to mimic the natural healing process of bones as a whole—the material strength required for the support of hard tissues, the degradation of the implanted biomaterials or medical devices, the release profile of the loaded drugs and the biological cues required for cell differentiation.

## Figures and Tables

**Table 1:** Existing calcium phosphates and their main properties [8].

Molar Ratio (Ca/P)	Compound	Formula	Solubility at 25 °C		pH Stability
			−Log (K_s_)	g L^−1^	
0.5	Monocalcium phosphate monohydrate (MCPM)	Ca(H_2_PO_4_)_2_*H_2_O	1.14	~18	0.0–2.0
0.5	Monocalcium phosphate anhydrous (MCPA or MCP)	Ca(H_2_PO_4_)_2_	1.14	~17	a
1.0	Dicalcium phosphate dihydrate (DCPD), mineral brushite	CaHPO_4_*2H_2_O	6.59	~0.088	2–6
1.0	Dicalcium phosphate anhydrous (DCPA or DCP), mineral monetite	CaHPO_4_	6.9	~0.048	a
1.33	Octacalcium phosphate (OCP)	Ca_8_(HPO_4_)_2_(PO_4_)_4_*5H_2_O	96.6	~0.0081	5.5–7.0
1.5	α-Tricalcium phosphate (α-TCP)	α-Ca_3_(PO_4_)_2_	25.5	~0.0025	b
1.5	β-Tricalcium phosphate (β-TCP)	β-Ca_3_(PO_4_)_2_	28.9	~0.0005	b
1.2–2.2	Amorphous calcium phosphate (ACP)	Ca_x_H_y_(PO_4_)_z_*nH_2_O, n = 3–4.5, 15–20% H_2_O	c	c	~5–12 d
1.5–1.67	Calcium-deficient hydroxyapatite (CDHA) e	Ca_10−x_(HPO_4_)_x_(PO_4_)_x_(OH)_2−x_ (0 < x < 1)	~85	~0.0094	6.5–9.5
1.67	Hydroxyapatite (HA, Hap or OHAp)	Ca_10_(PO_4_)_6_(OH)_2_	116.8	~0.0003	9.5–12
1.67	Fluorapatite (FA or Fap)	Ca_10_(PO_4_)_6_F_2_	120.0	~0.0002	7–12
1.67	Oxyapatite (OA, Oap or OXA) f	Ca_10_(PO_4_)_6_O	~69	~0.087	b
2.0	Tetracalcium phosphate (TTCP), mineral hilgenstockite	Ca_4_(PO_4_)_2_O	38–44	~0.0007	b

a: Stable at temperatures above 100 °C. b: These compounds cannot be precipitated from aqueous solutions. c: Cannot be measured precisely; however, the following values were found: 25.7 ± 0.1 (pH 7.40), 29.9 ± 0.1 (pH 6.00), 32.7 ± 0.1 (pH 5.28). The comparative extent of dissolution in acidic buffer is: ACP >> α-TCP >> β-TCP > CDHA >> HA > FA. d: Always metastable. e: Occasionally referred to as ‘‘precipitated HA’’ (PHA), f: The existence of OA remains questionable.

**Table 2:** MicroRNA for regulation of bone markers and their effect on targeted genes.

MicroRNA	Effect	Target Gene	Cells	Reference
miR-17-92	Promotes normal bone metabolism	RUNX2, type I collagen	MC3T3-E1 (mouse pre-osteoblasts cell line)	[101]
miR-125b	Inhibits proliferation and osteogenic differentiation	Unknown	hBMCs (human bone marrow cells)	[102]
miR-133a	Inhibits osteogenesis	RUNX2	C2C12 (mouse myoblast cell line)	[94]
miR-135	Inhibits osteogenesis	Smad-5	C2C12 (mouse myoblast cell line)	[94]
miR-196a	Promotes osteogenesis	RUNX2, OPN	BMSCs (bone marrow cells)	[103]
miR-2861	Promotes osteogenic differentiation	HDAC5	Mouse BMSs (mouse bone marrow cells)	[98]
antagomiR-133a	Increases osteogenesis	RUNX2	hMSCs (human mesenchymal stem cells)	[96]
